# Author Correction: Adaptation of *Plasmodium falciparum* to humans involved the loss of an ape-specific erythrocyte invasion ligand

**DOI:** 10.1038/s41467-021-21491-y

**Published:** 2021-02-16

**Authors:** William R. Proto, Sasha V. Siegel, Selasi Dankwa, Weimin Liu, Alison Kemp, Sarah Marsden, Zenon A. Zenonos, Steve Unwin, Paul M. Sharp, Gavin J. Wright, Beatrice H. Hahn, Manoj T. Duraisingh, Julian C. Rayner

**Affiliations:** 1 Malaria Programme, Wellcome Sanger Institute,Wellcome Genome Campus, Cambridge, CB10 1SA UK; 2 Department of Immunology and Infectious Diseases, Harvard T.H. Chan School of Public Health, Boston, MA USA; 3 Departments of Medicine and Microbiology, University of Pennsylvania, Philadelphia, PA 19104 USA; 4 Department of Microbiology, University of Pennsylvania, Philadelphia, PA 19104 USA; 5 Chester Zoo, Chester, CH2 1LH UK; 6 Institute of Evolutionary Biology, University of Edinburgh, Edinburgh, UK; 7 Present Address: School of Biosciences, University of Birmingham, Edgbaston, B15 2TT UK; 8 Present Address: Cambridge Institute for Medical Research, University of Cambridge, Hills Road, Cambridge, CB2 0XY UK

**Keywords:** Parasite biology, Parasite evolution, Malaria

Correction to: *Nature Communications* 10.1038/s41467-019-12294-3, published online 4 October 2019.

The original version of this Article contained an error in Figure 5c, which incorrectly displayed a region on chromosome 4, rather than the correct region on chromosome 11.

This has been corrected in both the PDF and HTML versions of the Article.

