# Supplementary material for: Adaptation of Plasmodium falciparum to humans involved the loss of an ape-specific erythrocyte invasion ligand
Source: Nat Commun. 2019 Oct 4;10:4512. doi: 10.1038/s41467-019-12294-3 (PMC6778099; doi:10.1038/s41467-019-12294-3)
Supplement: Supplementary file 1 — Supplementary Information [file 41467_2019_12294_MOESM1_ESM.pdf]

Supplementary Information

Adaptation of *Plasmodium falciparum* to humans involved the loss of an ape-specific erythrocyte invasion ligand

Proto et al

```
     1
Pf   ALCKLENYIKNEYDRENSFYILNNKVEKEYEKGT-NNNINIDKLANGSLEMLDK-KEALLEDKKNVDNKDIKEQLPENNK
G1   ALCKLENYIKNEYDRENSFYILNNKVEKEYEKGT-NNNINIDKLANGSLEMLEVKKEALLEDKKNVDNKDIKEQLPEHNK
G2   TLCKLENYLKNEYEKENSFNIINNKVEKEYDSGT-NNNVNIDSSSNGKLELLEVKEDALLND------KNIQEQEAENNI
C1   ALCKLENYIKNEYDKENSVYIINNKVEKDYGSGMNNNNINIDKLPNGSLEILKVKKEALLGDKKNIDNKDIKEQLPENNK
C2   TLCKLENYLKNEYEKENSFNIINNKVVKEYDSGT-NNNVNIDRSSNGRLELLEVKEEALLND------KNIEEEEAENNI
C3   TLCKLENNIKNKYDKENSFYIINNKVEKAYEGGT-NNNINIDKLSNGSLEMLEVKEETLFDDKKNIHNKDIKEQLTENNI
B1   ALCKLENYIKNEYES-----------------GM-NNNINIDKLPNGSLEILKVKKEALLGDKKNIDNKDIKEQLPENNK

    81
Pf   NFEEKILHEDICKLNKENIEKEGLYKPNTFKSIGNDLLYKKGKLNFLYEYGMELLHINKVPMNRMNNSRGLGDSNMSFLD
G1   NFEEKILYEDICKLNKENIEKEGLYKPNTFKSIGNDLLYKKGKLNFLYEYGMELLHINKVPMNRMNNSRGLGDSNMSFLD
G2   NFEEKILHKNVYELNKESIEKEGLYKPNTIKNIEDESLYKKGKLNFLYEYGMELLNINKVRMNKMNNSRGLGDSNTSFLD
C1   NFEEKILHKDICKLNKENIEKEGLYKPNTFKNIENDLLYKKGKLNFLYDYGMELLHINKAPMNRMNNSRGLGDGDMSFLD
C2   NFEEKILYKNVCELNKENIEKEGLYKPNTIKNIENDSLYKKGMLNFLYEYGMELLNINKVPMNKMNNSRVLGDSKMSFLD
C3   NYEEKNLLKDICILNKEKIEKEGLYKPSTFKYIENDSLYKKGKLNFLYVYGMELLHINKTSMNRMNNFRVLGDSNMSYLD
B1   NFEEKILHKDICKLNKENIENEGLYKPNTFKNIENDLLYKKGKLNFLYEYGMELLHINKARMNRMNNSRGLGDSNMSFLD

   161
Pf   ISNNNMNSLNSM---NNLNNSNNFDSSNSLSFVDMKSIHRCIKKRAKGERDWACNDKNTKEPNICVSDRRVQLCTGNLIE
G1   ISNNNMNSLNNLNNLNNLNNSNNFDSSNSLSFVDMKSIHRCIKKRAKGERDWACNDKNTKEPNICVSDRRVQLCTGNLIE
G2   ISNNNNKN-----------KNNNMNNLNSLSFVDMKSVHRCITKRKKGEKDWACNDKNTKEPNICVSDRRVQLCTGNLVD
C1   ISNNNMNISNSL-------------DSLNSLSFVDMKSIHRCIKKRAKGERDWVCNNKNTKEPNICVSDRKVQLCTGNLIE
C2   ISNNNNKN-----------KNNNMNNLNSLSFVDMKSIHRCITKRKKGEKDWACNDKNTKEPNICVSDRRVQLCTGNLVD
C3   ISNNTNN--NNMNSLNSSNNLNNMNSFNSLSFVDMKFLHRCIKKRNKGERDWKCNDINTKEPNICVSDRRVQLCTGNLID
B1   ISNNNMNILNSVDN----------LDSLNSLSFVDMKSIHRCIKKRAKGERDWVCNNKNTKEPNVCVSDRKVQLCTGNLIE

   241
Pf   LPINDSTKEKFKEKLILAAQKEGSLLFEKFGKKYNEEFCLNLKWSYGDYGDIIK
G1   LPINDSTKDKFKEKLILAAQKEGSLLFEKFGKKYNEEFCLNLKWSYGDYGDIIK
G2   LSIDESTKEKFKEKLIIAAKREGTLLFEKFGKEYTAEFCLNLKWSYSDYGDIIK
C1   LPISDSTKEKFKEKLILAAQKEGSLLFEKFGKKYNEEFCLNLKWSYGDYGDIIK
C2   LSINDATKEKFEGKLIIAAKREGTLLFEKFGKKYTAEFCLNLKWSYSDYGDIIK
C3   IPTNESTKEKFKEKLILAAKREGSLLFEKFGKIYNAEFCLNLKWSYSDYGDIIK
B1   LPISDSTKEKFKEKLILAAEKEGNLLFEKFGKKYNEEFCLNLKWSYGDYGDIIK
```

Supplementary Figure 1. PfEBA165 full alignment.

Exemplar EBA165 amino acid sequences (~800 bp amplified fragment) from ape-infective *Laverania* species (G1, *Plasmodium praefalciparum*; G2, *P. adleri*; G3, *P. blacklocki*; C1, *P. reichenowi*; C2, *P gaboni*; C3, *P. billcollinsi;* B1, *P. lomamiensis*) as recently described [3]. The 3D7 *P. falciparum* sequence shown is that which would be translated if both frameshifts were corrected, and represents the relevant fragment of the full-length corrected PfEBA165 protein ectodomain that was expressed recombinantly.

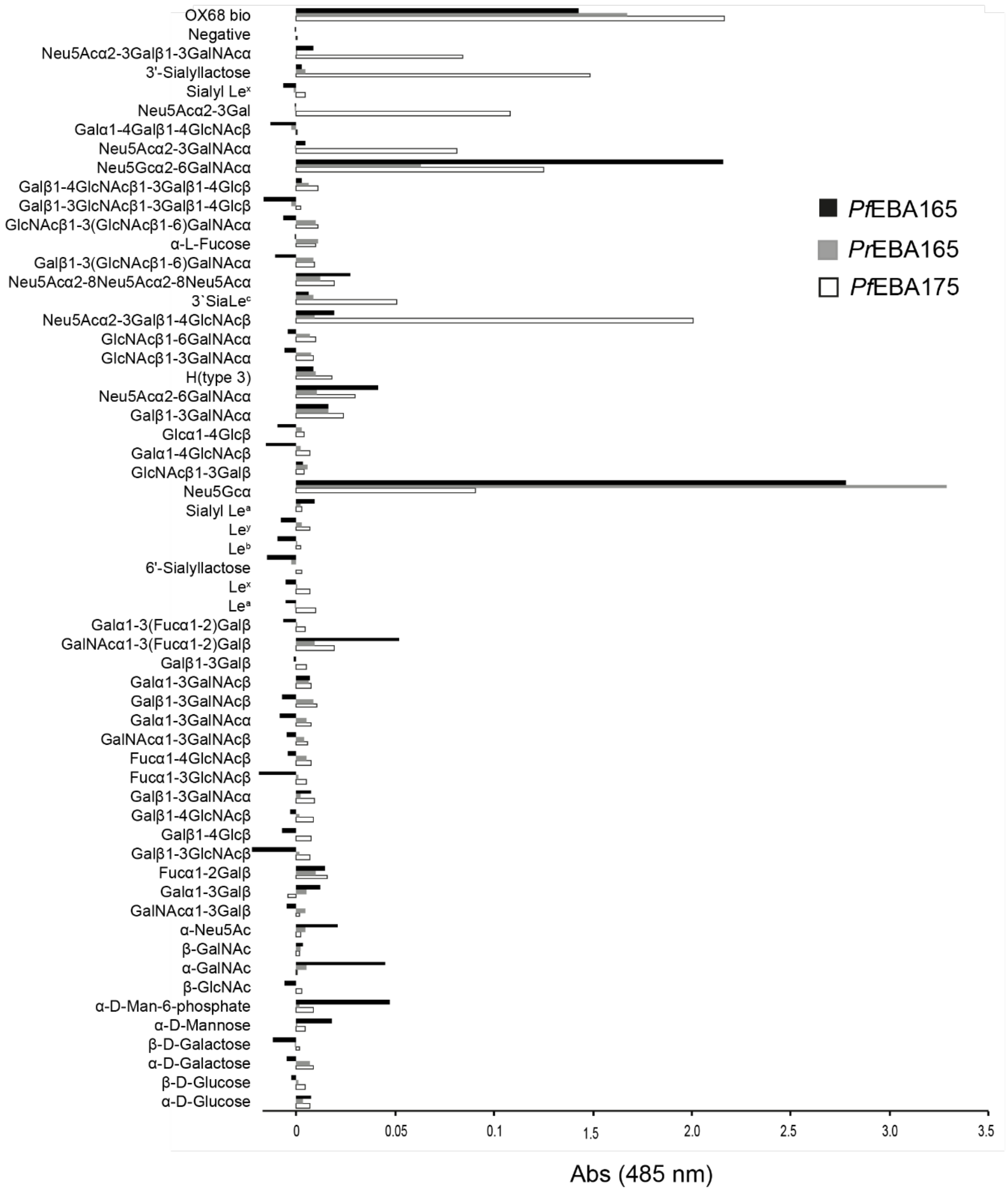

Supplementary Figure 2. PfEBA165, PrEBA165 and PfEBA175 bind primarily sialic acid containing glycans

Purified mono-biotinylated glycans (GlycoTech) were immobilised on streptavidin-coated 96 well plates and probed with normalised recombinant pentameric β-lactamase-tagged PfEBA165, PrEBA165 and PfEBA175 (black, grey, and white bars, respectively). Binding was detected by incubation with nitrocefin, a substrate for β-lactamase. As expected from

the known binding specificity of PfEBA175, most binding was detected with glycans containing sialic acids. Data points represent mean values of three technical replicates, conducted on the same batches of recombinant proteins and glycans.

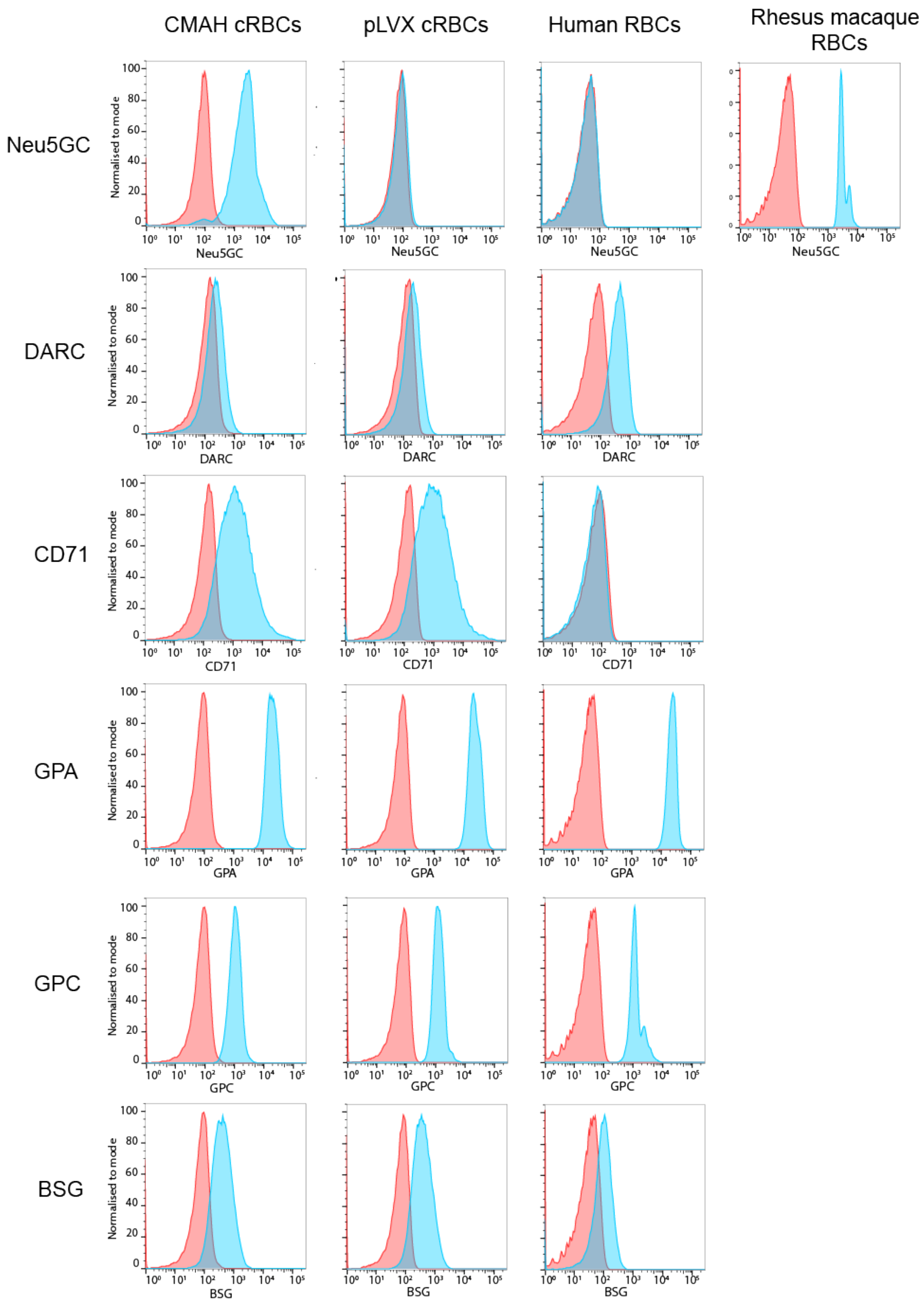

Supplementary Figure 3. CMAH expression has no impact on surface levels of major erythrocyte receptors.

Surface expression of Basigin (BSG), Glycophorin C (GPC), Glycophorin A (GPA), CD71, Duffy Antigen Receptor for Chemokines (DARC) was measured by incubating cultured red

blood cells (cRBCs) or human red blood cells (RBCs) with either commercial monoclonal antibodies followed by fluorescently tagged secondary antibodies (BSG and Neu5Gc), or primary antibodies directly conjugated to fluorophores (DARC, CD71, GPA, GPC), with binding detected by flow cytometry (blue traces). Secondary antibodies alone were used as a negative control for binding (red traces). There was no detectable difference in the surface expression of known *P. falciparum* invasion receptors (BSG, GPA, GPC) between cRBCs produced from hSCs transfected with control (pLVX) or *CMAH* expressing vectors. cRBCs expressed higher levels of CD71 and lower levels of DARC compared to human RBCs, as has been previously shown with the cRBC expression system, thought to reflect the fact that cRBCs represent more immature RBCs[1,2]. Expression of CMAH resulted in Neu5Gc expression on the cRBC surface. NeuGc is present in primate erythrocytes such as ape and macaque (shown), but not on human erythrocytes or control cRBCs.

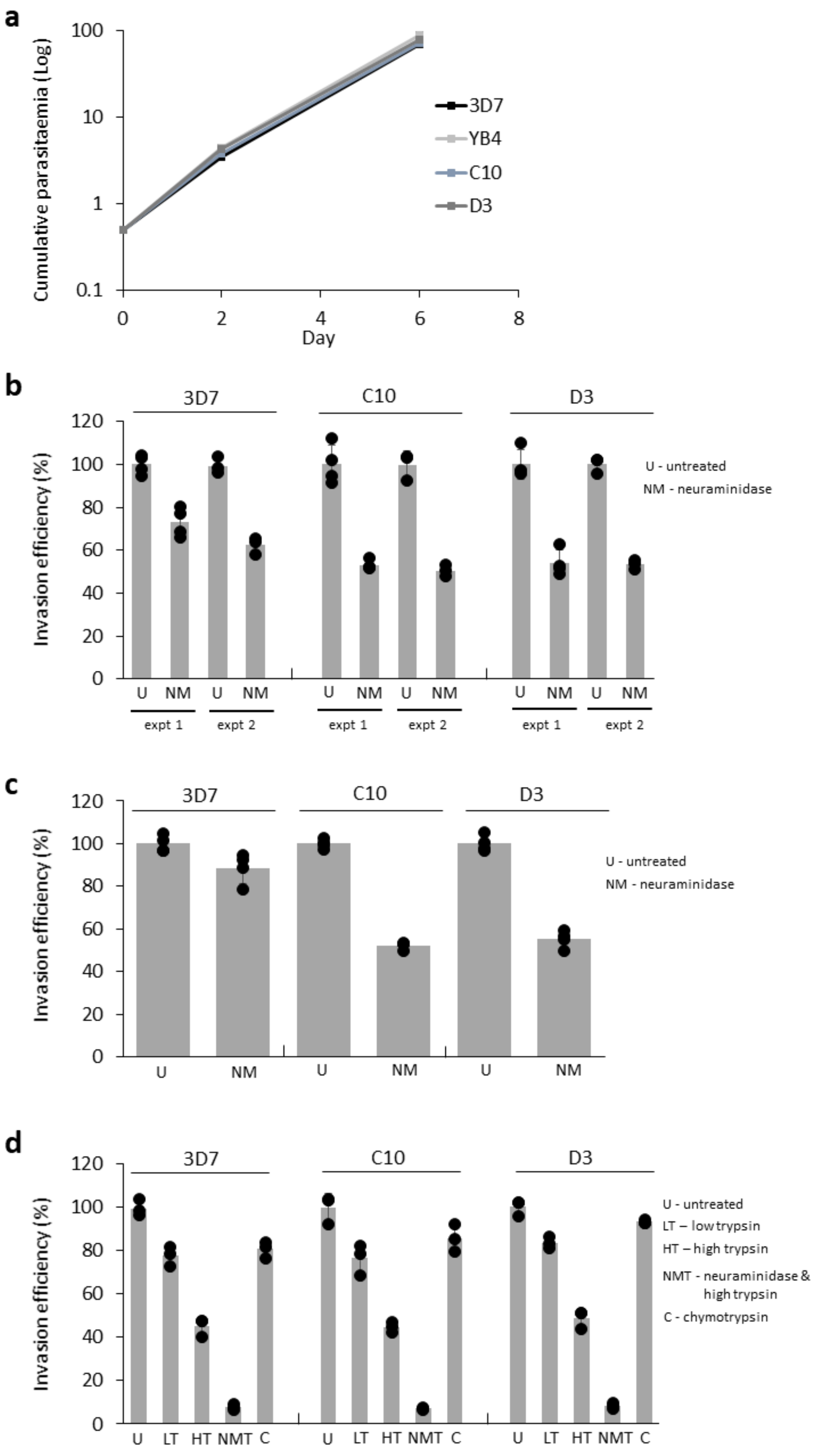

a
Cumulative parasitaemia (Log)
100
10
1
0.1
0
2
4
6
8
Day
3D7
YB4
C10
D3
b
3D7
C10
D3
Invasion efficiency (%)
120
100
80
60
40
20
0
U
NM
expt 1
expt 2
U - untreated
NM - neuraminidase
c
3D7
C10
D3
Invasion efficiency (%)
U
NM
U - untreated
NM - neuraminidase
d
3D7
C10
D3
Invasion efficiency (%)
U
LT
HT
NMT
C
U - untreated
LT – low trypsin
HT – high trypsin
NMT - neuraminidase & high trypsin
C - chymotrypsin

Supplementary Figure 4. Genome editing of PfEBA165 to correct frameshift mutations has no impact on erythrocyte invasion profile or *in vitro* growth rate.

A) Relative growth of *P. falciparum* 3D7 (parental), YB4 (cloned transfectant with 1 frameshift corrected) and C10 and D3 (cloned transfectant lines with both frameshifts corrected) lines during continuous culture in human erythrocytes. Lines were diluted with fresh red blood cells every two days, parasitaemia counted using flow cytometry, and cumulative parasitaemia calculated adjusting for the dilution factors. There was no difference in growth rates between the lines. Data points represents mean values of three biological replicates. Error bars represent standard deviation.

B) Invasion of *P. falciparum* 3D7 (parental), and C10 and D3 (cloned transfectant lines with both frameshifts corrected) into human erythrocytes that were either untreated (u), or treated with neuraminidase (NM) to remove surface sialic acids. Erythrocytes were labelled with fluorescent dyes, and new invasion events were identified using a two-colour flow cytometry assay[3]. Two separate assays were conducted (expt 1 and expt 2). Invasion rates are expressed relative to the invasion into untreated human erythrocytes of each strain in separate experiments. Bars represent mean values of four technical replicates for expt 1 and three technical replicates for expt 2, with individual data points overlaid as black circles. Error bars represent standard deviation.

C) Invasion of *P. falciparum* 3D7 (parental), and C10 and D3 (cloned transfectant lines with both frameshifts corrected) into chimpanzee erythrocytes that were either untreated (u), or treated with neuraminidase (NM) to remove surface sialic acids. Erythrocytes were labelled with fluorescent dyes, and new invasion events were identified using a two-colour flow cytometry assay[3]. Invasion rates are expressed relative to the invasion into untreated chimpanzee erythrocytes of each strain. Bars represent mean values of four technical replicates from one assay, with individual data points overlaid as black circles. Error bars represent standard deviation.

D) *P. falciparum* 3D7 (parental), and C10 and D3 (cloned transfectant lines with both frameshifts corrected) lines were incubated with enzyme treated erythrocytes for 24 hours,

and invasion measured using two-colour flow cytometry to count invasion only into treated erythrocytes. Data points represent mean values of three technical replicates from a single assay. Error bars represent standard deviation. Invasion rates are expressed relative to invasion into untreated erythrocytes for each parasite line.

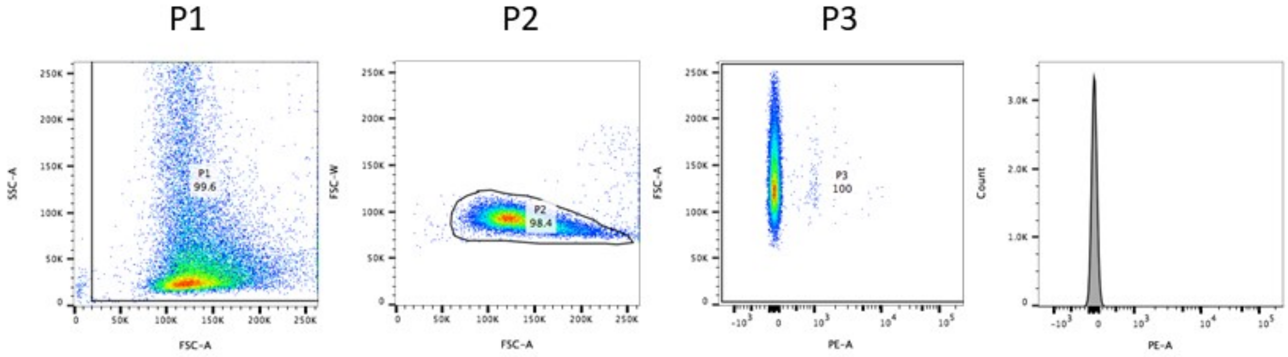

Supplementary Figure 5. Flow cytometry gating strategy for erythrocyte binding assays.

Gates were determined using control samples with cells only. Cells were selected using gates P1 (side scatter-area (SSC-A) vs forward scatter-area (FSC-A)), P2 (forward scatter-width (FSC-W) and forward scatter-area (FSC-A)), and P3 (forward scatter-area (FSC-A) and phycoerthrin-area (PE-A)).

Supplementary Table 1. Significantly down-regulated genes in *PfEBA165* corrected lines.

GeneID	Gene description	Log_2FC C10	p-value C10	Log_2FC D3	p-value D3
Pf3D7_0424200	Reticulocyte binding protein homologue (Rh4)	-1.03	3.32E-42	NA	NA
Pf3D7_0424300	Erythrocyte binding antigen 165 (EBA165)	-2.20	8.33E-137	-2.18	3.41E-78
Pf3D7_0424400	Surface-associated interspersed protein 4.2 (SURFIN 4.2)	-1.28	0.028	-1.78	0.003
Pf3D7_0424500	Serine/threonine protein kinase, FIKK family	-1.25	3.33E-14	-1.23	3.48E-10
Pf3D7_0424600	Plasmodium exported protein (PHISTb, unknown function)	-0.89	2.87E-26	-0.69	0.001
Pf3D7_0424700	Serine/threonine protein kinase, FIKK family	-0.65	7.81E-05	-1.44	1.16E-10
Pf3D7_0424800	Plasmodium exported protein (PHISTa, unknown function)	-2.27	0.0007	-1.84	0.003
Pf3D7_0424900	Plasmodium exported protein (PHISTb, unknown function)	-1.18	8.08E-08	-1.64	1.08E-08
Pf3D7_1147000	Sporozoite and liver stage asparagine-rich protein (SLARP)	-6.29	9.31E-234	-6.62	1.13E-159
Pf3D7_1147100	Dynein light chain, putative	-3.69	0.0006	-3.44	0.0004
Pf3D7_1147200	Tubulin tyrosine ligase, putative	-3.74	2.43E-110	-3.16	2.87E-54

Significantly down-regulated gene clusters in PfEBA165 corrected lines (double-edited clones C10 and D3). Genes that are significantly down-regulated (p-value ≤ 0.05) in both C10 and D3 relative to the parent line 3D7 in the order are listed in which they appear along the two down-regulated clusters of genes located on chromosomes 4 and 11, respectively.

Supplementary Table 2. Oligonucleotides used in this study

Oligo	Description	Gene name	Gene ID	5' to 3' sequence
OL176	gRNA 5' frameshift. Anneal to OL177.	*PfEAB165*	PF3D7_0424300	ATTGATAAGTTGGCAAATGGGAGTT
OL177	gRNA 5' frameshift. Anneal to OL176	*PfEAB165*	PF3D7_0424300	AAACAACTCCCATTTGCCAACTTAT
OL186	Phosphorothoiate modified oligo. Product for transfection, targets 5' frameshift.	*PfEAB165*	PF3D7_0424300	C*A*AGAAAGAAAGAAAAACAAGC
OL187	Phosphorothoiate modified oligo. Product for transfection, targets 5' frameshift.	*PfEBA165*	PF3D7_0424300	G*T*ACTCCCTTCTTTTTGTGC
OL231	gRNA 3' frameshift. Anneal to OL232.	*PfEAB165*	PF3D7_0424300	ATTGAACATAAAGAGAAATTTCCAA
OL232	gRNA 3' frameshift. Anneal to OL231.	*PfEAB165*	PF3D7_0424300	AAACTTGGAAATTTCTCTTTATGTT
OL257	Phosphorothoiate modified oligo. Product for transfection, targets 3' frameshift.	*PfEAB165*	PF3D7_0424300	C*A*AGAGAAATTGATTTTGGCAGC
OL258	Phosphorothoiate modified oligo. Product for transfection, targets 3' frameshift.	*PfEBA165*	PF3D7_0424300	G*T*TCAAGATACATATTCTCATGC
OL293	Amplify PCR product for sequencing	*PfEAB165*	PF3D7_0424300	TTAACCCTGATTTTGATGC
OL271	Amplify PCR product for sequencing	*PfEAB165*	PF3D7_0424300	TCTCTCCACATATCTTTACC
OL265	qRT-PCR	*PfAMA1*	PF3D7_1133400	CAGCTGCTGTCGCTGTATTA
OL266	qRT-PCR	*PfAMA1*	PF3D7_1133400	CCATAATCTTGTGGTTCATCCATTT
OL307	qRT-PCR	*PfEBA165*	PF3D7_0424300	AGGTGGCACACAAAGTAGTC
OL308	qRT-PCR	*PfEBA165*	PF3D7_0424300	CAGTGCTTGGATCTTCTCTACC
OL315	qRT-PCR	*Pfcyp87*	PF3D7_0510200	AAACGGGAGATCCTTCAGGT
OL316	qRT-PCR	*Pfcyp87*	PF3D7_0510200	AAGGACATGGGACAGTGGTT

References:

1. Dankwa, S., Lim, C., Bei, A. K., Jiang, R. H. Y., Abshire, J. R. et al. Ancient human sialic acid variant restricts an emerging zoonotic malaria parasite. *Nat. Commun.* **7,** 11187 (2016).
2. Giarratana, M. C., Rouard, H., Dumont, A., Kiger, L., Safeukui, I. et al. Proof of principle for transfusion of in vitro–generated red blood cells. *Blood* **118**, 5071-5079 (2011).
3. Theron, M., Hesketh, R. L., Subramanian, S. & Rayner, J. C. An adaptable two-color flow cytometric assay to quantitate the invasion of erythrocytes by *Plasmodium falciparum* parasites. *Cytometry A* **77**, 1067-1074, doi:10.1002/cyto.a.20972 (2010).
