# Supplementary material for: Adaptation of Plasmodium falciparum to humans involved the loss of an ape-specific erythrocyte invasion ligand
Source: Nat Commun. 2019 Oct 4;10:4512. doi: 10.1038/s41467-019-12294-3 (PMC6778099; doi:10.1038/s41467-019-12294-3)
Supplement: Supplementary file 3 — Reporting Summary [file 41467_2019_12294_MOESM3_ESM.pdf]

nature research

Corresponding author(s): Rayner, Julian C

Last updated by author(s): 13/09/2019

Reporting Summary

Nature Research wishes to improve the reproducibility of the work that we publish. This form provides structure for consistency and transparency in reporting. For further information on Nature Research policies, see Authors & Referees and the Editorial Policy Checklist .

Statistics

For all statistical analyses, confirm that the following items are present in the figure legend, table legend, main text, or Methods section.

n/a	Confirmed	
☐	☒	The exact sample size (*n*) for each experimental group/condition, given as a discrete number and unit of measurement
☐	☒	A statement on whether measurements were taken from distinct samples or whether the same sample was measured repeatedly
☐	☒	The statistical test(s) used AND whether they are one- or two-sided<br>*Only common tests should be described solely by name; describe more complex techniques in the Methods section.*
☒	☐	A description of all covariates tested
☒	☐	A description of any assumptions or corrections, such as tests of normality and adjustment for multiple comparisons
☒	☐	A full description of the statistical parameters including central tendency (e.g. means) or other basic estimates (e.g. regression coefficient) AND variation (e.g. standard deviation) or associated estimates of uncertainty (e.g. confidence intervals)
☒	☐	For null hypothesis testing, the test statistic (e.g. F, t, r) with confidence intervals, effect sizes, degrees of freedom and P value noted<br>*Give P values as exact values whenever suitable.*
☒	☐	For Bayesian analysis, information on the choice of priors and Markov chain Monte Carlo settings
☒	☐	For hierarchical and complex designs, identification of the appropriate level for tests and full reporting of outcomes
☒	☐	Estimates of effect sizes (e.g. Cohen's *d*, Pearson's *r*), indicating how they were calculated

Our web collection on statistics for biologists contains articles on many of the points above.

Software and code

Policy information about availability of computer code

Data collection	No software was used to collect data used in this study.
Data analysis	Flow cytometry analysis was performed using FlowJo version 10.0.7 and 10.0.8 (Tree Star).<br>Tophat2 (v2.1.1, default parameters) was used to map RNAseq paired end reads to the P. falciparum 3D7 genome (PlasmoDB v29), DESeq2 (v1.12.3) was used in the R statistical environment to normalize and determine significantly differentially expressed genes. For whole genome sequencing of P. falciparum CRISPR-edited lines, paired end reads were aligned using BWA-MEM (https://arxiv.org/abs/1303.3997) and variant detection was performed with Freebayes [https://arxiv.org/abs/1207.3907]. SnpEff (Cingolani et al., 2012) was used to predict the effect of detected variants on translated protein sequences. All software used in data analysis for this study are open source.

For manuscripts utilizing custom algorithms or software that are central to the research but not yet described in published literature, software must be made available to editors/reviewers. We strongly encourage code deposition in a community repository (e.g. GitHub). See the Nature Research guidelines for submitting code & software for further information.

Data

Policy information about availability of data

All manuscripts must include a data availability statement. This statement should provide the following information, where applicable:

- Accession codes, unique identifiers, or web links for publicly available datasets
- A list of figures that have associated raw data
- A description of any restrictions on data availability

RNA-sequencing data have been deposited in the European Nucleotide Archive (ENA) with the accession number ERP114933. The source data for Figs 2a, 3a, 4c and Supplementary Figs 4a-d , are available in the Source Data file. All other data are available from the authors upon reasonable request.

October 2018

Field-specific reporting

Please select the one below that is the best fit for your research. If you are not sure, read the appropriate sections before making your selection.

- [x] Life sciences
- [ ] Behavioural & social sciences
- [ ] Ecological, evolutionary & environmental sciences

For a reference copy of the document with all sections, see nature.com/documents/nr-reporting-summary-flat.pdf

Life sciences study design

All studies must disclose on these points even when the disclosure is negative.

Sample size	No sample size calculation was performed.
Data exclusions	No recorded data were excluded from the analyses.
Replication	To verify reproducibility of experimental findings, where possible the experiments were replicated. Data are presented as averages or as representative, and details are provided in the methods and legends
Randomization	For the analyses conducted in this study randomization was not used.
Blinding	Blinding was not relevant to this type of molecular analysis

Reporting for specific materials, systems and methods

We require information from authors about some types of materials, experimental systems and methods used in many studies. Here, indicate whether each material, system or method listed is relevant to your study. If you are not sure if a list item applies to your research, read the appropriate section before selecting a response.

Materials & experimental systems

n/a	Involved in the study	
	x	Antibodies
	x	Eukaryotic cell lines
x		Palaeontology
	x	Animals and other organisms
x		Human research participants
x		Clinical data

Methods

n/a	Involved in the study	
x		ChIP-seq
	x	Flow cytometry
x		MRI-based neuroimaging

Antibodies

Antibodies used	anti-Neu5Gc (1:5,000, Siamab), phycoerythrin(PE)-conjugated anti-DARC (1:10, Miltenyi Biotec), anti-CD71-PE (1:10, Miltenyi Biotec), anti-BSG (1:1,000, Clone MEM-M6/6, Axxora [Exbio]), fluorescein isothiocyanate-conjugated anti-glycophorin A (GPA) (1:50, Clone 2B7, STEMCELL Technologies) and anti-glycophorin C (GPC)-FITC (1:500, BRIC 10, Santa Cruz), streptavidin-horseradish peroxidase (1:2000, Cell Signaling Technology).
Validation	All antibodies were purchased from commercial providers; validation information provided by manufacturers.

Eukaryotic cell lines

Policy information about cell lines

Cell line source(s)	Wild type 3D7 Plasmodium falciparum strain was obtained from Malaria Research and Reference Reagent Resource Centre (www.mr4.org).
Authentication	CRISPR-edited lines were generated as described, and authenticated by locus specific genotyping and whole genome sequencing.
Mycoplasma contamination	Parasite lines were not tested for mycoplasma contamination.
Commonly misidentified lines (See ICLAC register)	N/A

Animals and other organisms

Policy information about studies involving animals; ARRIVE guidelines recommended for reporting animal research

Laboratory animals	Study did not involve lab animals
Wild animals	Study did not involve wild animals
Field-collected samples	Field samples were not collected for this study
Ethics oversight	The only animal material used in the study was discarded chimpanzee material, left over after collection for health testing at Chester Zoo, UK.

Note that full information on the approval of the study protocol must also be provided in the manuscript.

Flow Cytometry

Plots

Confirm that:

- [x] The axis labels state the marker and fluorochrome used (e.g. CD4-FITC).
- [x] The axis scales are clearly visible. Include numbers along axes only for bottom left plot of group (a 'group' is an analysis of identical markers).
- [ ] All plots are contour plots with outliers or pseudocolor plots.
- [ ] A numerical value for number of cells or percentage (with statistics) is provided.

Methodology

Sample preparation	The assay was modified from previously published protocols (Crosnier et al., 2013; Petersen et al., 2011). Briefly, monomeric biotinylated PfEBA165, PrEBA165 and PfEBA175 protein supernatants were immobilised on 0.4-0.6 µm streptavidin coated Nile Red fluorescent microbeads(Spherotech), by incubation with gentle agitation at 4oC for 1 h. Loaded microbeadswere washed with Buffer A (HBS, 1% BSA) and incubated in an ice-cold sonicating water bath for 20 min to disrupt aggregates. Binding reactions were carried out in flat-bottomed 96-well plates, with each well containing ~4 x 10^5 cells and loaded fluorescent microbeads, mixed at estimated cell to fluorescent bead ratio of 1:145. After 1 h at 4oC with gentle agitation, cells were washed twice in Buffer A and analysed by flow cytometry.
Instrument	LSRII cytometer (BD Biosciences) for erythrocytes and MACSQuant (Miltenyi Biotech) for cultured red blood cells (cRBCs).
Software	FlowJo version 10.0.7 and 10.0.8 (Tree Star).
Cell population abundance	Flow cytometry analyses did not include cell sorting.
Gating strategy	Gating strategy determined using control samples with cells only. Gates: P1 SSC-A/FSC-A; P2 FSC-W/FSC-A; P3 FSC-A/PE-A.

- [x] Tick this box to confirm that a figure exemplifying the gating strategy is provided in the Supplementary Information.
